# Supplementary material for: Antimicrobial Susceptibility of Bacterial Pathogens from Patients with Ocular Surface Infections in Germany, 2020–2021: A Comparison with the Data from Three Previous National Studies
Source: Antibiotics (Basel). 2024 May 21;13(6):471. doi: 10.3390/antibiotics13060471 (PMC11200635; doi:10.3390/antibiotics13060471)
Supplement: Supplementary file 1 [file antibiotics-13-00471-s001.zip › antibiotics-3000881-supplementary.pdf]

Table S1: Bacterial isolates and patient demographic data

Designation	Study			
	OS1	OS2	OS3	OS4
Year	2004	2009	2015	2020-2021
Collection period	June - November (6 months)	April - September (6 months)	February - September (8 months)	April '20 - December '21 (21 months)
Study sites (n)	35	31	27	20
Isolates analyzed (n)	1 108	1 156	1 110	1 366
Isolates per study site (range; median)	10-125; 35	9-121; 34	19-72; 39	26-162; 64
Bacterial species / group (n; %)				
Staphylococcus aureus	436; 39.4	395; 34.2	360; 32.4	594; 43.5
Streptococcus pneumoniae	187; 16.9	212; 18.3	240; 21.6	149; 10.9
Haemophilus influenzae	164; 14.8	234; 20.2	325; 29.5	178; 13.0
Enterobacterales	164; 14.8	129; 11.2	not tested	235; 17.3
Pseudomonas aeruginosa	44; 4.0	36; 3.1	34; 3.1	64; 4.7
Staphylococcus epidermidis	80; 7.2	91; 7.9	101; 9.1	70; 5.1
Moraxella catarrhalis	33; 3.0	59; 5.1	50; 4.5	76; 5.6
Patients (n)	1 108	1 156	1 110	1 366
Patient care (n; %):				
Outpatient	749; 67.6	786; 68.0	785; 70.7	919; 67.3
Inpatient	359; 32.4	370; 32.0	268; 24.1	268; 19.6
Not specified	-	-	57; 5.1	179; 13.1
Patient gender (n; %):				
Female	538; 48.6	545; 47.1	538; 48.5	671; 49.1
Male	543; 49.0	599; 51.8	571; 51.4	695; 50.9
Not specified	27; 2.4	12; 1.0	1; 0.1	-
Patient age (n; %):				
≤ 1 year	471; 42.5	461; 39.9	515; 46.4	612; 44.8
2-9 years	138; 12.5	219; 18.9	184; 16.6	141; 10.3
10-19 years	43; 3.9	35; 3.0	20; 1.8	29; 2.1
20-39 years	72; 6.5	65; 5.6	69; 6.2	70; 5.1
40-59 years	124; 11.2	101; 8.7	88; 7.9	126; 9.2
60-79 years	177; 16.0	162; 14.0	146; 13.2	216; 15.8
≥ 80 years	78; 7.0	110; 9.5	88; 7.9	170; 12.4
Not specified	5; 0.5	3; 0.3	none	2; 0.1
Range (years)	< 1-97	< 1-97	< 1-96	< 1-99
Median (years)	4	3	2	3
Specimen type (n; %):				
Eye smear without further specification	216; 19.5	258; 22.3	241; 27.7	547; 40.0
Eye secretion	none	none	1; 0.09	9; 0.7
Conjunctival smear	673; 60.7	810: 70.1	821; 74.0	742; 54.3
Corneal smear	28; 2.5	30; 2.6	24; 2.2	19; 1.4
Eyelid smear	29; 2.6	29; 2.5	14; 1.3	44; 3.2
Other sampling material	26; 2.3	29; 2.5	9; 0.8	5; 0.4

Not specified	136; 12.3	none	none	none
Type of infection (n; %)				
Blepharitis	26; 2.3	3; 0.3	4; 0.4	18; 1.3
Hordeolum	7; 0.6	1; 0.1	5; 0.5	5; 0.4
Conjunctivitis	524; 47.3	461; 39.9	534; 48.1	639; 46.8
Keratitis	28; 2.5	8; 0.7	13; 1.2	13; 1.0
Keratoconjunctivitis	13; 1.2	10; 0.9	7; 0.6	10; 0.7
Others	44; 4.0	49; 4.2	78; 7.0	89; 6.5
Not specified	477; 43.1	624; 54.0	469; 42.3	592; 43.3

Table S2: Cut-off values (effective 11-30-23)

Antibiotic	Species	MIC (mg/l)		Source (EUCAST)
		WS	PR	
Chloramphenicol	*S. aureus*	≤ 16	> 16	ECOFF (**7**)
	S. epidermidis	Not specified		
	S. pneumoniae	≤ 8	> 8	ECOFF (**7**)
	H. influenzae	≤ 2	> 2	ECOFF (**7**)
	M. catarrhalis	≤ 2	> 2	Screening cut-off v.13.1 (**6**)
	P. aeruginosa	Not specified		
	Enterobacterales	≤ 16	> 16	Screening cut-off v.13.1 (**6**)
Gentamicin	*S. aureus*	≤ 2	> 2	ECOFF (**7**)
	S. epidermidis	≤ 0.25	> 0.25	(T)ECOFF (**7**)
	S. pneumoniae	Not specified		
	H. influenzae	≤ 4	> 4	Screening cut-off v.13.1 (**6**)
	M. catarrhalis	Not specified		
	P. aeruginosa	≤ 8	> 8	ECOFF (**7**)
	Enterobacterales	≤ 2	> 2	Screening cut-off v.13.1 (**6**)
Kanamycin	*Staphylococcus aureus*	≤ 8	> 8	(T)ECOFF (**7**)
	Staphylococcus epidermidis	Not specified		
	Streptococcus pneumoniae	Not specified		
	Haemophilus influenzae	Not specified		
	Moraxella catarrhalis	Not specified		
	Pseudomonas aeruginosa	≤ 256	> 256	ECOFF (**7**)
	Enterobacterales	Not specified		
Neomycin	*Staphylococcus aureus*	≤ 1	> 1	(T)ECOFF (**7**)
	Staphylococcus epidermidis	Not specified		
	Streptococcus pneumoniae	Not specified		
	Haemophilus influenzae	Not specified		
	Moraxella catarrhalis	Not specified		
	Pseudomonas aeruginosa	≤ 64	> 64	(T)ECOFF (**7**)
	Enterobacterales	≤ 8	> 8	Screening cut-off v.13.1 (**6**)
Levofloxacin	*Staphylococcus aureus*	≤ 1	> 1	ECOFF (**7**)
	Staphylococcus epidermidis	≤ 0.5	> 0.5	ECOFF (**7**)
	Streptococcus pneumoniae	≤ 2	> 2	ECOFF (**7**)
	Haemophilus influenzae	≤ 0.063	> 0.063	ECOFF (**7**)
	Moraxella catarrhalis	≤ 0.125	> 0.125	ECOFF (**7**)
	Pseudomonas aeruginosa	≤ 2	> 2	ECOFF (**7**)
	Enterobacterales	≤ 0.25	> 0.25	Screening cut-off v.13.1 (**6**)
Ofloxacin	*Staphylococcus aureus*	≤ 1	> 1	ECOFF (**7**)
	Staphylococcus epidermidis	Not specified		
	Streptococcus pneumoniae	≤ 4	> 4	ECOFF (**7**)
	Haemophilus influenzae	≤ 0.063	> 0.063	ECOFF (**7**)
	Moraxella catarrhalis	≤ 0.25	> 0.25	(T)ECOFF (**7**)
	Pseudomonas aeruginosa	≤ 4	> 4	ECOFF (**7**)
	Enterobacterales	≤ 0.25	> 0.25	Screening cut-off v.13.1 (**6**)
Oxytetracycline	*Staphylococcus aureus*	≤ 2	> 2	presumable (T)ECOFF (**7**)[1]
	Staphylococcus epidermidis	≤ 2	> 2	presumable (T)ECOFF (**7**)[1]
	Streptococcus pneumoniae	≤ 2	> 2	presumable (T)ECOFF (**7**)[1]
	Haemophilus influenzae	≤ 2	> 2	presumable (T)ECOFF (**7**)[1]
	Moraxella catarrhalis	≤ 4	> 4	presumable (T)ECOFF (**7**)[1]
	Pseudomonas aeruginosa	≤ 128	> 128	presumable (T)ECOFF (**7**)[1]
	Enterobacterales	Not specified		
Cefoxitin	*Staphylococcus aureus*	≤ 4	> 4	Screening cut-off v.13.1 (**6**)
Oxacillin	*Staphylococcus aureus*	≤ 2	> 2	Screening cut-off v.13.1 (**6**)
	Staphylococcus epidermidis	≤ 0.25	> 0.25	Screening cut-off v.13.1 (**6**)

WS, wildtype susceptible; PR, phenotypically resistant

[1]EUCAST has not defined cut-off-values for oxytetracycline.

Table S3: *In-vitro* activity of the tested antibacterial agents against *S. aureus* isolates obtained from patients with ocular surface infections (n=594)

Substance		MIC (mg/l)																	MIC_{50} (mg/l)	MIC_{90} (mg/l)	cut-off value (mg/l)[1]		S	R	n
		≤ 0.008	0.016	0.031	0.063	0.125	0.25	0.5	1	2	4	8	16	32	64	128	256	≥512							
Cefoxitin	abs.	/	/	/	/	/	/	*0*	1	198	375	0	4	**16**	/	/	/	/	4	4	Screening Cut-off (4)	n	574	20	594
	cum %	/	/	/	/	/	/	*0.0*	0.2	33.5	96.6	96.6	97.3	**100.0**	/	/	/	/				%	96.6	3.4	
Oxacillin	abs.	/	/	/	/	/	*378*	182	14	1	0	3	**16**	/	/	/	/	/	*0.25*	0.5	Screening Cut-off (2)	n	575	19	594
	cum %	/	/	/	/	/	*63.6*	94.3	96.6	96.8	96.8	97.3	**100.0**	/	/	/	/	/				%	96.8	3.2	
Chloramphenicol	abs.	/	/	/	/	*0*	0	0	0	4	65	506	17	0	1	1	0	**0**	8	8	ECOFF (16)	n	592	2	594
	cum %	/	/	/	/	*0.0*	0.0	0.0	0.0	0.7	11.6	96.8	99.7	99.7	99.8	100.0	100.0	**100.0**				%	99.7	0.3	
Gentamicin	abs.	/	/	*0*	0	4	238	292	46	3	1	0	2	3	3	**2**	/	/	0.5	1	ECOFF (2)	n	583	11	594
	cum %	/	/	*0.0*	0.0	0.7	40.7	89.9	97.6	98.1	98.3	98.3	98.7	99.2	99.7	**100.0**	/	/				%	98.1	1.9	
Kanamycin	abs.	/	/	/	/	*0*	0	2	42	392	116	20	2	1	1	4	5	**9**	2	4	(T)ECOFF (8)	n	572	22	594
	cum %	/	/	/	/	*0.0*	0.0	0.3	7.4	73.4	92.9	96.3	96.6	96.8	97.0	97.6	98.5	**100.0**				%	96.3	3.7	
Neomycin	abs.	/	/	/	/	*2*	73	406	91	12	0	1	1	5	1	0	0	**2**	0.5	1	(T)ECOFF (1)	n	572	22	594
	cum %	/	/	/	/	*0.3*	12.6	81.0	96.3	98.3	98.3	98.5	98.7	99.5	99.7	99.7	99.7	**100.0**				%	96.3	3.7	
Levofloxacin	abs.	*0*	0	0	1	53	423	69	4	2	3	4	14	**21**	/	/	/	/	0.25	0.5	ECOFF (1)	n	550	44	594
	cum %	*0.0*	0.0	0.0	0.2	9.1	80.3	91.9	92.6	92.9	93.4	94.1	96.5	**100.0**	/	/	/	/				%	92.6	7.4	
Ofloxacin	abs.	/	*0*	0	0	2	124	401	22	1	2	3	4	**35**	/	/	/	/	0.5	1	ECOFF (1)	n	549	45	594
	cum %	/	*0.0*	0.0	0.0	0.3	21.2	88.7	92.4	92.6	92.9	93.4	94.1	**100.0**	/	/	/	/				%	92.4	7.6	
Oxytetracycline	abs.	/	/	*0*	0	1	6	245	311	15	1	0	0	1	1	**13**	/	/	1	1	(T)ECOFF (2)[2]	n	578	16	594
	cum %	/	/	*0.0*	0.0	0.2	1.2	42.4	94.8	97.3	97.5	97.5	97.5	97.6	97.8	**100.0**	/	/				%	97.3	2.7	

[1]According to the specifications on the EUCAST website "Distributions and ECOFFs" (**7**) and the specifications for topically applied antimicrobial substances according to the EUCAST document "Breakpoint tables for interpretation of MICs and zone diameters" (**6**).

[2]EUCAST has not defined breakpoints / cut-off values for oxytetracyline. The ECOFF of tetracycline is 1 mg/l. The (T)ECOFF of oxytetracycline can be assumed to be either 1 mg/l or 2 mg/l.

abs., absolute; cum %, cumulated frequency in percent; /, concentration not tested; S, (wildtype) susceptible; R, resistant / acquired resistance mechanism; ECOFF, epidemiological cut-off value; (T)ECOFF, tentative ECOFF

Values presented in italics indicate the number and percentage of strains that display MICs lower or equivalent to the lowest concentration tested

Values presented in bold indicate the number and percentage of strains that display MICs higher or equivalent to the highest concentration tested

Table S4: *In-vitro* activity of the tested antibacterial agents against cefoxitin(methicillin)-susceptible *S. aureus* isolates obtained from patients with ocular surface infections (n=574)

Substance		MIC (mg/l)																	MIC_{50} (mg/l)	MIC_{90} (mg/l)	cut-off value (mg/l)[1]		S	R	n
		≤ 0.008	0.016	0.031	0.063	0.125	0.25	0.5	1	2	4	8	16	32	64	128	256	≥512							
Cefoxitin	abs.	/	/	/	/	/	/	*0*	1	198	375	0	0	**0**	/	/	/	/	4	4	ECOFF (4)	n	574	0	574
	cum %	/	/	/	/	/	/	*0.0*	0.2	34.7	100.0	100.0	100.0	**100.0**	/	/	/	/				%	100.0	0.0	
Oxacillin	abs.	/	/	/	/	/	*378*	182	14	0	0	0	**0**	/	/	/	/	/	*0.25*	0.5	ECOFF (2)	n	574	0	574
	cum %	/	/	/	/	/	*65.9*	97.6	100.0	100.0	100.0	100.0	**100.0**	/	/	/	/	/				%	100.0	0.0	
Chloramphenicol	abs.	/	/	/	/	*0*	0	0	0	4	62	490	16	0	1	1	0	**0**	8	8	ECOFF (16)	n	572	2	574
	cum %	/	/	/	/	*0.0*	0.0	0.0	0.0	0.7	11.5	96.9	99.7	99.7	99.8	100.0	100.0	**100.0**				%	99.7	0.3	
Gentamicin	abs.	/	/	*0*	0	4	233	280	44	3	0	0	2	3	3	**2**	/	/	0.5	0.5	ECOFF (2)	n	564	10	574
	cum %	/	/	*0.0*	0.0	0.7	41.3	90.1	97.7	98.3	98.3	98.3	98.6	99.1	99.7	**100.0**	/	/				%	98.3	1.7	
Kanamycin	abs.	/	/	/	/	*0*	0	2	41	382	113	20	1	0	1	3	5	**6**	2	4	(T)ECOFF (8)	n	558	16	574
	cum %	/	/	/	/	*0.0*	0.0	0.3	7.5	74.0	93.7	97.2	97.4	97.4	97.6	98.1	99.0	**100.0**				%	97.2	2.8	
Neomycin	abs.	/	/	/	/	*1*	72	395	90	11	0	0	1	3	0	0	0	**1**	0.5	1	(T)ECOFF (1)	n	558	16	574
	cum %	/	/	/	/	*0.2*	12.7	81.5	97.2	99.1	99.1	99.1	99.3	99.8	99.8	99.8	99.8	**100.0**				%	97.2	2.8	
Levofloxacin	abs.	*0*	0	0	1	53	414	65	4	2	2	4	12	**17**	/	/	/	/	0.25	0.5	ECOFF (1)	n	537	37	574
	cum %	*0.0*	0.0	0.0	0.2	9.4	81.5	92.9	93.6	93.9	94.3	94.9	97.0	**100.0**	/	/	/	/				%	93.6	6.4	
Ofloxacin	abs.	/	*0*	0	0	2	123	391	20	1	2	2	4	**29**	/	/	/	/	0.5	1	ECOFF (1)	n	536	38	574
	cum %	/	*0.0*	0.0	0.0	0.3	21.8	89.9	93.4	93.6	93.9	94.3	94.9	**100.0**	/	/	/	/				%	93.4	6.6	
Oxytetracycline	abs.	/	/	*0*	0	1	6	240	300	14	0	0	0	1	1	**11**	/	/	1	1	(T)ECOFF (2)[2]	n	561	13	574
	cum %	/	/	*0.0*	0.0	0.2	1.2	43.0	95.3	97.7	97.7	97.7	97.7	97.9	98.1	**100.0**	/	/				%	97.7	2.3	

[1]According to the specifications on the EUCAST website "Distributions and ECOFFs" (**7**) and the specifications for topically applied antimicrobial substances according to the EUCAST document "Breakpoint tables for interpretation of MICs and zone diameters" (**6**).

[2]EUCAST has not defined breakpoints / cut-off values for oxytetracyline. The ECOFF of tetracycline is 1 mg/l. The (T)ECOFF of oxytetracycline can be assumed to be either 1 mg/l or 2 mg/l.

abs., absolute; cum %, cumulated frequency in percent; /, concentration not tested; S, (wildtype) susceptible; R, resistant / acquired resistance mechanism; ECOFF, epidemiological cut-off value; (T)ECOFF, tentative ECOFF

Values presented in italics indicate the number and percentage of strains that display MICs lower or equivalent to the lowest concentration tested

Values presented in bold indicate the number and percentage of strains that display MICs higher or equivalent to the highest concentration tested

Table S5*: In-vitro* activity of the tested antibacterial agents against cefoxitin(methicillin)-resistant *S. aureus* isolates obtained from patients with ocular surface infections (n=20)

Substance		MIC (mg/l) ≤ 0.008	0.016	0.031	0.063	0.125	0.25	0.5	1	2	4	8	16	32	64	128	256	≥512	MIC_{50} (mg/l)	MIC_{90} (mg/l)	cut-off value (mg/l)[1]		S	R	n
Cefoxitin	abs.	/	/	/	/	/	/	*0*	0	0	0	0	4	**16**	/	/	/	/	**32**	**32**	ECOFF (4)	n	0	20	20
	cum %	/	/	/	/	/	/	*0.0*	0.0	0.0	0.0	0.0	20.0	**100.0**	/	/	/	/				%	0.0	100.0	
Oxacillin	abs.	/	/	/	/	/	*0*	0	0	1	0	3	**16**	/	/	/	/	/	**16**	**16**	ECOFF (2)	n	1	19	20
	cum %	/	/	/	/	/	*0.0*	0.0	0.0	5.0	5.0	20.0	**100.0**	/	/	/	/	/				%	5.0	95.0	
Chloramphenicol	abs.	/	/	/	/	*0*	0	0	0	0	3	16	1	0	0	0	0	**0**	8	8	ECOFF (16)	n	20	0	20
	cum %	/	/	/	/	*0.0*	0.0	0.0	0.0	0.0	15.0	95.0	100.0	100.0	100.0	100.0	100.0	**100.0**				%	100.0	0.0	
Gentamicin	abs.	/	/	*0*	0	0	5	12	2	0	1	0	0	0	0	**0**	/	/	0.5	1	ECOFF (2)	n	19	1	20
	cum %	/	/	*0.0*	0.0	0.0	25.0	85.0	95.0	95.0	100.0	100.0	100.0	100.0	100.0	**100.0**	/	/				%	95.0	5.0	
Kanamycin	abs.	/	/	/	/	*0*	0	0	1	10	3	0	1	1	0	1	0	**3**	2	**≥512**	(T)ECOFF (8)	n	14	6	20
	cum %	/	/	/	/	*0.0*	0.0	0.0	5.0	55.0	70.0	70.0	75.0	80.0	80.0	85.0	85.0	**100.0**				%	70.0	30.0	
Neomycin	abs.	/	/	/	/	*1*	1	11	1	1	0	1	0	2	1	0	0	**1**	0.5	32	(T)ECOFF (1)	n	14	6	20
	cum %	/	/	/	/	*5.0*	10.0	65.0	70.0	75.0	75.0	80.0	80.0	90.0	95.0	95.0	95.0	**100.0**				%	70.0	30.0	
Levofloxacin	abs.	*0*	0	0	0	0	9	4	0	0	1	0	2	**4**	/	/	/	/	0.5	**32**	ECOFF (1)	n	13	7	20
	cum %	*0.0*	0.0	0.0	0.0	0.0	45.0	65.0	65.0	65.0	70.0	70.0	80.0	**100.0**	/	/	/	/				%	65.0	35.0	
Ofloxacin	abs.	/	*0*	0	0	0	1	10	2	0	0	1	0	**6**	/	/	/	/	0.5	**32**	ECOFF (1)	n	13	7	20
	cum %	/	*0.0*	0.0	0.0	0.0	5.0	55.0	65.0	65.0	65.0	70.0	70.0	**100.0**	/	/	/	/				%	65.0	35.0	
Oxytetracycline	abs.	/	/	*0*	0	0	0	5	11	1	1	0	0	0	0	**2**	/	/	1	4	(T)ECOFF (2)[2]	n	17	3	20
	cum %	/	/	*0.0*	0.0	0.0	0.0	25.0	80.0	85.0	90.0	90.0	90.0	90.0	90.0	**100.0**	/	/				%	85.0	15.0	

[1]According to the specifications on the EUCAST website "Distributions and ECOFFs" (**7**) and the specifications for topically applied antimicrobial substances according to the EUCAST document "Breakpoint tables for interpretation of MICs and zone diameters" (**6**).

[2]EUCAST has not defined breakpoints / cut-off values for oxytetracyline. The ECOFF of tetracycline is 1 mg/l. The (T)ECOFF of oxytetracycline can be assumed to be either 1 mg/l or 2 mg/l.

abs., absolute; cum %, cumulated frequency in percent; /, concentration not tested; S, (wildtype) susceptible; R, resistant / acquired resistance mechanism; ECOFF, epidemiological cut-off value; (T)ECOFF, tentative ECOFF

Values presented in italics indicate the number and percentage of strains that display MICs lower or equivalent to the lowest concentration tested.

Values presented in bold indicate the number and percentage of strains that display MICs higher or equivalent to the highest concentration tested

Table S6: *In-vitro* activity of the tested antibacterial agents against *S. epidermidis* isolates obtained from patients with ocular surface infections (n=70)

Substance		MIC (mg/l) ≤ 0.008	0.016	0.031	0.063	0.125	0.25	0.5	1	2	4	8	16	32	64	128	256	≥512	MIC_{50} (mg/l)	MIC_{90} (mg/l)	cut-off value (mg/l)[1]		S	R	n
Cefoxitin	abs.	/	/	/	/	/	/	*3*	10	32	5	3	6	**11**	/	/	/	/	2	**32**	ECOFF (4)	n	50	20	70
	cum %	/	/	/	/	/	/	*4.3*	18.6	64.3	71.4	75.7	84.3	**100.0**	/	/	/	/				%	71.4	28.6	
Oxacillin	abs.	/	/	/	/	/	*47*	2	1	3	3	4	**10**	/	/	/	/	/	*0.25*	**16**	Screening Cut-off (0.25)	n	47	23	70
	cum %	/	/	/	/	/	*67.1*	70.0	71.4	75.7	80.0	85.7	**100.0**	/	/	/	/	/				%	67.1	32.9	
Chloramphenicol	abs.	/	/	/	/	*0*	0	0	1	8	46	14	0	0	1	0	0	**0**	4	8	Not specified	n	-	-	70
	cum %	/	/	/	/	*0.0*	0.0	0.0	1.4	12.9	78.6	98.6	98.6	98.6	100.0	100.0	100.0	**100.0**				%	-	-	
Gentamicin	abs.	/	/	*0*	13	24	14	2	0	0	1	3	5	7	1	**0**	/	/	0.125	32	(T)ECOFF (0.25)	n	51	19	70
	cum %	/	/	*0.0*	18.6	52.9	72.9	75.7	75.7	75.7	77.1	81.4	88.6	98.6	100.0	**100.0**	/	/				%	72.9	27.1	
Kanamycin	abs.	/	/	/	/	*1*	3	25	16	2	4	2	0	2	2	4	5	**4**	1	256	Not specified	n	-	-	70
	cum %	/	/	/	/	*1.4*	5.7	41.4	64.3	67.1	72.9	75.7	75.7	78.6	81.4	87.1	94.3	**100.0**				%	-	-	
Neomycin	abs.	/	/	/	/	*38*	15	6	6	3	0	0	2	0	0	0	0	**0**	*0.125*	1	Not specified	n	-	-	70
	cum %	/	/	/	/	*54.3*	75.7	84.3	92.9	97.1	97.1	97.1	100.0	100.0	100.0	100.0	100.0	**100.0**				%	-	-	
Levofloxacin	abs.	*0*	0	0	0	1	41	5	0	4	13	5	1	**0**	/	/	/	/	0.25	4	ECOFF (0.5)	n	47	23	70
	cum %	*0.0*	0.0	0.0	0.0	1.4	60.0	67.1	67.1	72.9	91.4	98.6	100.0	**100.0**	/	/	/	/				%	67.1	32.9	
Ofloxacin	abs.	/	*0*	0	0	0	6	41	0	0	4	14	5	**0**	/	/	/	/	0.5	8	Not specified	n	-	-	70
	cum %	/	*0.0*	0.0	0.0	0.0	8.6	67.1	67.1	67.1	72.9	92.9	100.0	**100.0**	/	/	/	/				%	-	-	
Oxytetracycline	abs.	/	/	*0*	0	0	3	18	18	2	13	5	1	4	0	**6**	/	/	1	32	(T)ECOFF (2)[2]	n	41	29	70
	cum %	/	/	*0.0*	0.0	0.0	4.3	30.0	55.7	58.6	77.1	84.3	85.7	91.4	91.4	**100.0**	/	/				%	58.6	41.4	

[1]According to the specifications on the EUCAST website "Distributions and ECOFFs" (**7**) and the specifications for topically applied antimicrobial substances according to the EUCAST document "Breakpoint tables for interpretation of MICs and zone diameters" (**6**).

[2]EUCAST has not defined breakpoints / cut-off values for oxytetracyline. The (T)ECOFF of tetracycline is 1 mg/l. The (T)ECOFF of oxytetracycline can be assumed to be 2 mg/l.

abs., absolute; cum %, cumulated frequency in percent; /, concentration not tested; S, (wildtype) susceptible; R, resistant / acquired resistance mechanism; ECOFF, epidemiological cut-off value; (T)ECOFF, tentative ECOFF

Values presented in italics indicate the number and percentage of strains that display MICs lower or equivalent to the lowest concentration tested

Values presented in bold indicate the number and percentage of strains that display MICs higher or equivalent to the highest concentration tested

Table S7: *In-vitro* activity of the tested antibacterial agents against *S. pneumoniae* isolates obtained from patients with ocular surface infections (n=149)

Substance		MIC (mg/l)																	MIC$_{50}$ (mg/l)	MIC$_{90}$ (mg/l)	cut-off value (mg/l)[1]		S	R	n
		≤ 0.008	0.016	0.031	0.063	0.125	0.25	0.5	1	2	4	8	16	32	64	128	256	≥512							
Cefoxitin	abs.	/	/	/	/	/	/	*6*	51	52	10	13	11	**6**	/	/	/	/	2	16	Not specified	n	-	-	149
	cum %	/	/	/	/	/	/	*4.0*	38.3	73.2	79.9	88.6	96.0	**100.0**	/	/	/	/				%	-	-	
Oxacillin	abs.	/	/	/	/	/	*105*	7	5	15	2	7	**8**	/	/	/	/	/	*0.25*	8	Not specified	n	-	-	149
	cum %	/	/	/	/	/	*70.5*	75.2	78.5	88.6	89.9	94.6	**100.0**	/	/	/	/	/				%	-	-	
Chloramphenicol	abs.	/	/	/	/	*0*	0	0	9	98	37	5	0	0	0	0	0	**0**	2	4	ECOFF (8)	n	149	0	149
	cum %	/	/	/	/	*0.0*	0.0	0.0	6.0	71.8	96.6	100.0	100.0	100.0	100.0	100.0	100.0	**100.0**				%	100.0	0.0	
Gentamicin	abs.	/	/	*0*	0	0	0	1	2	10	45	87	4	0	0	**0**	/	/	8	8	Not specified	n	-	-	149
	cum %	/	/	*0.0*	0.0	0.0	0.0	0.7	2.0	8.7	38.9	97.3	100.0	100.0	100.0	**100.0**	/	/				%	-	-	
Kanamycin	abs.	/	/	/	/	*0*	0	0	0	0	1	3	18	72	54	0	0	**1**	32	64	Not specified	n	-	-	149
	cum %	/	/	/	/	*0.0*	0.0	0.0	0.0	0.0	0.7	2.7	14.8	63.1	99.3	99.3	99.3	**100.0**				%	-	-	
Neomycin	abs.	/	/	/	/	*0*	0	1	0	0	0	8	13	49	66	11	0	**1**	64	64	Not specified	n	-	-	149
	cum %	/	/	/	/	*0.0*	0.0	0.7	0.7	0.7	0.7	6.0	14.8	47.7	91.9	99.3	99.3	**100.0**				%	-	-	
Levofloxacin	abs.	*0*	0	0	0	0	1	31	112	5	0	0	0	**0**	/	/	/	/	1	1	ECOFF (2)	n	149	0	149
	cum %	*0.0*	0.0	0.0	0.0	0.0	0.7	21.5	96.6	100.0	100.0	100.0	100.0	**100.0**	/	/	/	/				%	100.0	0.0	
Ofloxacin	abs.	/	*0*	0	0	0	0	2	34	111	2	0	0	**0**	/	/	/	/	2	2	ECOFF (4)	n	149	0	149
	cum %	/	*0.0*	0.0	0.0	0.0	0.0	1.3	24.2	98.7	100.0	100.0	100.0	**100.0**	/	/	/	/				%	100.0	0.0	
Oxytetracycline	abs.	/	/	*0*	0	17	98	22	3	3	1	1	0	1	2	**1**	/	/	0.25	0.5	(T)ECOFF (2)[2]	n	143	6	149
	cum %	/	/	*0.0*	0.0	11.4	77.2	91.9	94.0	96.0	96.6	97.3	97.3	98.0	99.3	**100.0**	/	/				%	96.0	4.0	

[1]According to the specifications on the EUCAST website "Distributions and ECOFFs" (**7**) and the specifications for topically applied antimicrobial substances according to the EUCAST document "Breakpoint tables for interpretation of MICs and zone diameters" (**6**).

[2]EUCAST has not defined breakpoints / cut-off values for oxytetracyline. The ECOFF of tetracycline is 1 mg/l. The (T)ECOFF of oxytetracycline can be assumed to be either 1 mg/l or 2 mg/l.

abs., absolute; cum %, cumulated frequency in percent; /, concentration not tested; S, (wildtype) susceptible; R, resistant / acquired resistance mechanism; ECOFF, epidemiological cut-off value; (T)ECOFF, tentative ECOFF

Values presented in italics indicate the number and percentage of strains that display MICs lower or equivalent to the lowest concentration tested

Values presented in bold indicate the number and percentage of strains that display MICs higher or equivalent to the highest concentration tested

Table S8: *In-vitro* activity of the tested antibacterial agents against *H. influenzae* isolates obtained from patients with ocular surface infections (n=178)

Substance		MIC (mg/l)																	MIC$_{50}$ (mg/l)	MIC$_{90}$ (mg/l)	cut-off value (mg/l)[1]		S	R	n
		≤ 0.008	0.016	0.031	0.063	0.125	0.25	0.5	1	2	4	8	16	32	64	128	256	≥512							
Chloramphenicol	abs.	/	/	/	/	*9*	45	95	23	3	1	2	0	0	0	0	0	**0**	0.5	1	ECOFF (2)	n	175	3	178
	cum %	/	/	/	/	*5.1*	30.3	83.7	96.6	98.3	98.9	100.0	100.0	100.0	100.0	100.0	100.0	**100.0**				%	98.3	1.7	
Gentamicin	abs.	/	/	*0*	0	1	4	95	75	3	0	0	0	0	0	**0**	/	/	0.5	1	Screening Cut-off (4)	n	178	0	178
	cum %	/	/	*0.0*	0.0	0.6	2.8	56.2	98.3	100.0	100.0	100.0	100.0	100.0	100.0	**100.0**	/	/				%	100.0	0.0	
Kanamycin	abs.	/	/	/	/	*0*	1	0	21	133	19	1	1	0	0	0	0	**2**	2	4	Not specified	n	-	-	178
	cum %	/	/	/	/	*0.0*	0.6	0.6	12.4	87.1	97.8	98.3	98.9	98.9	98.9	98.9	98.9	**100.0**				%	-	-	
Neomycin	abs.	/	/	/	/	*0*	2	2	50	120	0	2	0	0	0	1	0	**1**	2	2	Not specified	n	-	-	178
	cum %	/	/	/	/	*0.0*	1.1	2.2	30.3	97.8	97.8	98.9	98.9	98.9	98.9	99.4	99.4	**100.0**				%	-	-	
Levofloxacin	abs.	*1*	113	55	2	1	0	2	1	1	0	1	0	**1**	/	/	/	/	0.016	0.031	ECOFF (0.063)	n	171	7	178
	cum %	*0.6*	64.0	94.9	96.1	96.6	96.6	97.8	98.3	98.9	98.9	99.4	99.4	**100.0**	/	/	/	/				%	96.1	3.9	
Ofloxacin	abs.	/	*7*	133	31	0	1	0	3	0	1	0	1	**1**	/	/	/	/	0.031	0.063	ECOFF (0.063)	n	171	7	178
	cum %	/	*3.9*	78.7	96.1	96.1	96.6	96.6	98.3	98.3	98.9	98.9	99.4	**100.0**	/	/	/	/				%	96.1	3.9	
Oxytetracycline	abs.	/	/	*0*	0	4	72	97	2	0	0	0	0	0	0	**3**	/	/	0.5	0.5	(T)ECOFF (2)[2]	n	175	3	178
	cum %	/	/	*0.0*	0.0	2.2	42.7	97.2	98.3	98.3	98.3	98.3	98.3	98.3	98.3	**100.0**	/	/				%	98.3	1.7	

[1]According to the specifications on the EUCAST website "Distributions and ECOFFs" (**7**) and the specifications for topically applied antimicrobial substances according to the EUCAST document "Breakpoint tables for interpretation of MICs and zone diameters" (**6**).

[2]EUCAST has not defined breakpoints / cut-off values for oxytetracyline. The ECOFF of tetracycline is 1 mg/l. The (T)ECOFF of oxytetracycline can be assumed to be either 1 mg/l or 2 mg/l.

abs., absolute; cum %, cumulated frequency in percent; /, concentration not tested; S, (wildtype) susceptible; R, resistant / acquired resistance mechanism; ECOFF, epidemiological cut-off value; (T)ECOFF, tentative ECOFF

Values presented in italics indicate the number and percentage of strains that display MICs lower or equivalent to the lowest concentration tested

Values presented in bold indicate the number and percentage of strains that display MICs higher or equivalent to the highest concentration tested

Table S9: *In-vitro* activity of the tested antibacterial agents against *M. catarrhalis* isolates obtained from patients with ocular surface infections (n=76)

Substance		MIC (mg/l)																	MIC_{50} (mg/l)	MIC_{90} (mg/l)	cut-off value (mg/l)[1]		S	R	n
		≤ 0.008	0.016	0.031	0.063	0.125	0.25	0.5	1	2	4	8	16	32	64	128	256	≥512							
Chloramphenicol	abs.	/	/	/	/	*0*	2	70	3	1	0	0	0	0	0	0	0	**0**	0.5	0.5	Screening Cut-off (2)	n	76	0	76
	cum %	/	/	/	/	*0.0*	2.6	94.7	98.7	100.0	100.0	100.0	100.0	100.0	100.0	100.0	100.0	**100.0**				%	100.0	0.0	
Gentamicin	abs.	/	/	*0*	1	55	20	0	0	0	0	0	0	0	0	**0**	/	/	0.125	0.25	Not specified	n	-	-	76
	cum %	/	/	*0.0*	1.3	73.7	100.0	100.0	100.0	100.0	100.0	100.0	100.0	100.0	100.0	**100.0**	/	/				%	-	-	
Kanamycin	abs.	/	/	/	/	*0*	2	48	26	0	0	0	0	0	0	0	0	**0**	0.5	1	Not specified	n	-	-	76
	cum %	/	/	/	/	*0.0*	2.6	65.8	100.0	100.0	100.0	100.0	100.0	100.0	100.0	100.0	100.0	**100.0**				%	-	-	
Neomycin	abs.	/	/	/	/	*7*	66	1	2	0	0	0	0	0	0	0	0	**0**	0.25	0.25	Not specified	n	-	-	76
	cum %	/	/	/	/	*9.2*	96.1	97.4	100.0	100.0	100.0	100.0	100.0	100.0	100.0	100.0	100.0	**100.0**				%	-	-	
Levofloxacin	abs.	*0*	0	4	69	3	0	0	0	0	0	0	0	**0**	/	/	/	/	0.063	0.063	ECOFF (0.125)	n	76	0	76
	cum %	*0.0*	0.0	5.3	96.1	100.0	100.0	100.0	100.0	100.0	100.0	100.0	100.0	**100.0**	/	/	/	/				%	100.0	0.0	
Ofloxacin	abs.	/	*0*	1	6	67	2	0	0	0	0	0	0	**0**	/	/	/	/	0.125	0.125	(T)ECOFF (0.25)	n	76	0	76
	cum %	/	*0.0*	1.3	9.2	97.4	100.0	100.0	100.0	100.0	100.0	100.0	100.0	**100.0**	/	/	/	/				%	100.0	0.0	
Oxytetracycline	abs.	/	/	*0*	0	1	29	42	4	0	0	0	0	0	0	**0**	/	/	0.5	0.5	(T)ECOFF (4)[2]	n	76	0	76
	cum %	/	/	*0.0*	0.0	1.3	39.5	94.7	100.0	100.0	100.0	100.0	100.0	100.0	100.0	**100.0**	/	/				%	100.0	0.0	

[1]According to the specifications on the EUCAST website "Distributions and ECOFFs" (**7**) and the specifications for topically applied antimicrobial substances according to the EUCAST document "Breakpoint tables for interpretation of MICs and zone diameters" (**6**).

[2]EUCAST has not defined breakpoints / cut-off values for oxytetracyline. The ECOFF of tetracycline is 2 mg/l. The (T)ECOFF of oxytetracycline can be assumed to be either 2 mg/l or 4 mg/l.

abs., absolute; cum %, cumulated frequency in percent; /, concentration not tested; S, (wildtype) susceptible; R, resistant / acquired resistance mechanism; ECOFF, epidemiological cut-off value; (T)ECOFF, tentative ECOFF

Values presented in italics indicate the number and percentage of strains that display MICs lower or equivalent to the lowest concentration tested

Values presented in bold indicate the number and percentage of strains that display MICs higher or equivalent to the highest concentration tested

Table S10: *In-vitro* activity of the tested antibacterial agents against *P. aeruginosa* isolates obtained from patients with ocular surface infections (n=64)

Substance		MIC (mg/l)																	MIC_{50} (mg/l)	MIC_{90} (mg/l)	cut-off value (mg/l)[1]		S	R	n
		≤ 0.008	0.016	0.031	0.063	0.125	0.25	0.5	1	2	4	8	16	32	64	128	256	≥512							
Chloramphenicol	abs.	/	/	/	/	*0*	0	0	0	0	0	0	0	1	31	24	6	**2**	64	256	Not specified	n	-	-	64
	cum %	/	/	/	/	*0.0*	0.0	0.0	0.0	0.0	0.0	0.0	0.0	1.6	50.0	87.5	96.9	**100.0**				%	-	-	
Gentamicin	abs.	/	/	*0*	0	0	1	1	28	28	3	1	0	0	0	**2**	/	/	2	2	ECOFF (8)	n	62	2	64
	cum %	/	/	*0.0*	0.0	0.0	1.6	3.1	46.9	90.6	95.3	96.9	96.9	96.9	96.9	**100.0**	/	/				%	96.9	3.1	
Kanamycin	abs.	/	/	/	/	*0*	0	0	1	0	0	1	2	8	25	23	3	**1**	64	128	ECOFF (256)	n	63	1	64
	cum %	/	/	/	/	*0.0*	0.0	0.0	1.6	1.6	1.6	3.1	6.3	18.8	57.8	93.8	98.4	**100.0**				%	98.4	1.6	
Neomycin	abs.	/	/	/	/	*0*	1	0	2	3	19	17	18	4	0	0	0	**0**	8	16	(T)ECOFF (64)	n	64	0	64
	cum %	/	/	/	/	*0.0*	1.6	1.6	4.7	9.4	39.1	65.6	93.8	100.0	100.0	100.0	100.0	**100.0**				%	100.0	0.0	
Levofloxacin	abs.	*0*	0	0	0	1	5	41	13	2	1	0	1	**0**	/	/	/	/	0.5	1	ECOFF (2)	n	62	2	64
	cum %	*0.0*	0.0	0.0	0.0	1.6	9.4	73.4	93.8	96.9	98.4	98.4	100.0	**100.0**	/	/	/	/				%	96.9	3.1	
Ofloxacin	abs.	/	*0*	0	0	0	2	17	37	5	2	0	0	**1**	/	/	/	/	1	2	ECOFF (4)	n	63	1	64
	cum %	/	*0.0*	0.0	0.0	0.0	3.1	29.7	87.5	95.3	98.4	98.4	98.4	**100.0**	/	/	/	/				%	98.4	1.6	
Oxytetracycline	abs.	/	/	*0*	0	0	0	0	0	0	1	9	32	22	0	**0**	/	/	16	32	(T)ECOFF (128)[2]	n	64	0	64
	cum %	/	/	*0.0*	0.0	0.0	0.0	0.0	0.0	0.0	1.6	15.6	65.6	100.0	100.0	**100.0**	/	/				%	100.0	0.0	

[1]According to the specifications on the EUCAST website "Distributions and ECOFFs" (**7**).

[2]EUCAST has not defined breakpoints / cut-off values for oxytetracyline. The ECOFF of tetracycline is 64 mg/l. The (T)ECOFF of oxytetracycline can be assumed to be either 64 mg/l or 128 mg/l.

abs., absolute; cum %, cumulated frequency in percent; /, concentration not tested; S, (wildtype) susceptible; R, resistant / acquired resistance mechanism; ECOFF, epidemiological cut-off value; (T)ECOFF, tentative ECOFF

Values presented in italics indicate the number and percentage of strains that display MICs lower or equivalent to the lowest concentration tested.

Values presented in bold indicate the number and percentage of strains that display MICs higher or equivalent to the highest concentration tested

Table S11*: In-vitro* activity of the tested antibacterial agents against Enterobacterales isolates obtained from patients with ocular surface infections (n=235)[a)]

Substance		MIC (mg/l)																	MIC_{50} (mg/l)	MIC_{90} (mg/l)	cut-off value (mg/l)[1]		S	R	n
		≤ 0.008	0.016	0.031	0.063	0.125	0.25	0.5	1	2	4	8	16	32	64	128	256	≥512							
Chloramphenicol	abs.	/	/	/	/	*0*	0	0	2	21	77	98	22	8	5	2	0	**0**	8	16	Screening Cut-off (16)	n	220	15	235
	cum %	/	/	/	/	*0.0*	0.0	0.0	0.9	9.8	42.6	84.3	93.6	97.0	99.1	100.0	100.0	**100.0**				%	93.6	6.4	
Gentamicin	abs.	/	/	*0*	0	1	102	99	22	3	3	0	4	0	1	**0**	/	/	0.5	1	Screening Cut-off (2)	n	227	8	235
	cum %	/	/	*0.0*	0.0	0.4	43.8	86.0	95.3	96.6	97.9	97.9	99.6	99.6	100.0	**100.0**	/	/				%	96.6	3.4	
Kanamycin	abs.	/	/	/	/	*0*	0	4	89	104	22	7	2	1	1	2	1	**2**	2	4	Not specified	n	-	-	235
	cum %	/	/	/	/	*0.0*	0.0	1.7	39.6	83.8	93.2	96.2	97.0	97.4	97.9	98.7	99.1	**100.0**				%	-	-	
Neomycin	abs.	/	/	/	/	*0*	7	128	70	21	5	1	2	1	0	0	0	**0**	0.5	2	Screening Cut-off (8)	n	232	3	235
	cum %	/	/	/	/	*0.0*	3.0	57.4	87.2	96.2	98.3	98.7	99.6	100.0	100.0	100.0	100.0	**100.0**				%	98.7	1.3	
Levofloxacin	abs.	*0*	4	43	118	33	17	9	3	2	0	3	3	**0**	/	/	/	/	0.063	0.25	Screening Cut-off (0.25)	n	215	20	235
	cum %	*0.0*	1.7	20.0	70.2	84.3	91.5	95.3	96.6	97.4	97.4	98.7	100.0	**100.0**	/	/	/	/				%	91.5	8.5	
Ofloxacin	abs.	/	*0*	6	64	102	32	16	4	4	1	1	2	**3**	/	/	/	/	0.125	0.5	Screening Cut-off (0.25)	n	204	31	235
	cum %	/	*0.0*	2.6	29.8	73.2	86.8	93.6	95.3	97.0	97.4	97.9	98.7	**100.0**	/	/	/	/				%	86.8	13.2	
Oxytetracycline	abs.	/	/	*0*	0	0	0	2	16	65	69	15	9	5	3	**51**	/	/	4	**128**	Not specified	n	-	-	235
	cum %	/	/	*0.0*	0.0	0.0	0.0	0.9	7.7	35.3	64.7	71.1	74.9	77.0	78.3	**100.0**	/	/				%	-	-	

[1]According to the specifications for topically applied antimicrobial substances according to the EUCAST document "Breakpoint tables for interpretation of MICs and zone diameters" (**6**).

abs., absolute; cum %, cumulated frequency in percent; /, concentration not tested; S, (wildtype) susceptible; R, resistant / acquired resistance mechanism; ECOFF, epidemiological cut-off value; (T)ECOFF, tentative ECOFF

Values presented in italics indicate the number and percentage of strains that display MICs lower or equivalent to the lowest concentration tested.

Values presented in bold indicate the number and percentage of strains that display MICs higher or equivalent to the highest concentration tested

[a)]Enterobacterales isolates belonged to the following species: *Escherichia coli* (n=50), *Enterobacter cloacae* complex (n=32), *Klebsiella pneumoniae* (n=30), *Serratia marcescens* (n=27), *Proteus mirabilis* (n=23), *Klebsiella oxytoca* (n=22), *Citrobacter koseri* (n=12), *Morganella morganii* (n=11), *Klebsiella aerogenes* (n=8), *Pantoea agglomerans* (n=4), *Proteus vulgaris* (n=3), *Pantoea* species (n=2), *Raoultella ornithinolytica* (n=2), *Citrobacter farmeri* (n=1), *Citrobacter freundii* (n=1), *Erwinia rhapontici* (n=1), *Hafnia alvei* (n=1), *Leclercia adecarboxylata* (n=1), *Pseudoescherichia vulneris* (n=1), *Raoultella planticola* (n=1), *Serratia liquefaciens* (n=1), *Serratia ureilytica* (n=1).

Table S12: *In-vitro* activity of the tested substances against *E. coli* isolates obtained from patients with ocular surface infections (n=50)

Substance		MIC (mg/l)																	MIC_{50} (mg/l)	MIC_{90} (mg/l)	cut-off value (mg/l)[1]		S	R	n
		≤ 0.008	0.016	0.031	0.063	0.125	0.25	0.5	1	2	4	8	16	32	64	128	256	≥512							
Chloramphenicol	abs.	/	/	/	/	*0*	0	0	0	3	17	29	0	0	1	0	0	**0**	8	8	ECOFF (16)	n	49	1	50
	cum %	/	/	/	/	*0.0*	0.0	0.0	0.0	6.0	40.0	98.0	98.0	98.0	100.0	100.0	100.0	**100.0**				%	98.0	2.0	
Gentamicin	abs.	/	/	*0*	0	0	9	29	7	0	1	0	3	0	1	**0**	/	/	0.5	1	ECOFF (2)	n	45	5	50
	cum %	/	/	*0.0*	0.0	0.0	18.0	76.0	90.0	90.0	92.0	92.0	98.0	98.0	100.0	**100.0**	/	/				%	90.0	10.0	
Kanamycin	abs.	/	/	/	/	*0*	0	0	5	32	7	3	0	1	1	0	0	**1**	2	8	(T)ECOFF (16)	n	47	3	50
	cum %	/	/	/	/	*0.0*	0.0	0.0	10.0	74.0	88.0	94.0	94.0	96.0	98.0	98.0	98.0	**100.0**				%	94.0	6.0	
Neomycin	abs.	/	/	/	/	*0*	0	18	22	8	1	0	1	0	0	0	0	**0**	1	2	ECOFF (8)	n	49	1	50
	cum %	/	/	/	/	*0.0*	0.0	36.0	80.0	96.0	98.0	98.0	100.0	100.0	100.0	100.0	100.0	**100.0**				%	98.0	2.0	
Levofloxacin	abs.	*0*	0	20	18	0	1	4	1	0	0	3	3	**0**	/	/	/	/	0.063	8	ECOFF (0.125)	n	38	12	50
	cum %	*0.0*	0.0	40.0	76.0	76.0	78.0	86.0	88.0	88.0	88.0	94.0	100.0	**100.0**	/	/	/	/				%	76.0	24.0	
Ofloxacin	abs.	/	*0*	1	24	13	0	3	2	1	0	1	2	**3**	/	/	/	/	0.063	8	ECOFF (0.25)	n	38	12	50
	cum %	/	*0.0*	2.0	50.0	76.0	76.0	82.0	86.0	88.0	88.0	90.0	94.0	**100.0**	/	/	/	/				%	76.0	24.0	
Oxytetracycline	abs.	/	/	*0*	0	0	0	0	3	21	15	0	0	0	0	**11**	/	/	4	**128**	(T)ECOFF (16)[2]	n	39	11	50
	cum %	/	/	*0.0*	0.0	0.0	0.0	0.0	6.0	48.0	78.0	78.0	78.0	78.0	78.0	**100.0**	/	/				%	78.0	22.0	

[1]According to the specifications on the EUCAST website "Distributions and ECOFFs" (**7**).

[2]EUCAST has not defined breakpoints / cut-off values for oxytetracyline. The ECOFF of tetracycline is 8 mg/l. The (T)ECOFF of oxytetracycline can be assumed to be either 8 mg/l or 16 mg/l.

abs., absolute; cum %, cumulated frequency in percent; /, concentration not tested; S, (wildtype) susceptible; R, resistant / acquired resistance mechanism; ECOFF, epidemiological cut-off value; (T)ECOFF, tentative ECOFF

Values presented in italics indicate the number and percentage of strains that display MICs lower or equivalent to the lowest concentration tested

Values presented in bold indicate the number and percentage of strains that display MICs higher or equivalent to the highest concentration tested

Table S13: *In-vitro* activity of the tested substances against *K. pneumoniae* isolates sampled from patients with ocular surface infections (n=30)

**Substance**		**MIC (mg/l)**																	**MIC_{50} (mg/l)**	**MIC_{90} (mg/l)**	**cut-off value (mg/l)[1]**		**S**	**R**	**n**
		**≤ 0.008**	**0.016**	**0.031**	**0.063**	**0.125**	**0.25**	**0.5**	**1**	**2**	**4**	**8**	**16**	**32**	**64**	**128**	**256**	**≥512**							
Chloramphenicol	abs.	/	/	/	/	*0*	0	0	0	1	21	6	1	0	1	0	0	**0**	4	8	Not specified	n	-	-	30
	cum %	/	/	/	/	*0.0*	0.0	0.0	0.0	3.3	73.3	93.3	96.7	96.7	100.0	100.0	100.0	**100.0**				%	-	-	
Gentamicin	abs.	/	/	*0*	0	0	20	9	0	0	0	0	1	0	0	**0**	/	/	0.25	0.5	ECOFF (2)	n	29	1	30
	cum %	/	/	*0.0*	0.0	0.0	66.7	96.7	96.7	96.7	96.7	96.7	100.0	100.0	100.0	**100.0**	/	/				%	96.7	3.3	
Kanamycin	abs.	/	/	/	/	*0*	0	2	20	4	2	0	0	0	0	1	0	**1**	1	4	(T)ECOFF (4)	n	28	2	30
	cum %	/	/	/	/	*0.0*	0.0	6.7	73.3	86.7	93.3	93.3	93.3	93.3	93.3	96.7	96.7	**100.0**				%	93.3	6.7	
Neomycin	abs.	/	/	/	/	*0*	3	24	1	0	1	1	0	0	0	0	0	**0**	0.5	0.5	(T)ECOFF (2)	n	28	2	30
	cum %	/	/	/	/	*0.0*	10.0	90.0	93.3	93.3	96.7	100.0	100.0	100.0	100.0	100.0	100.0	**100.0**				%	93.3	6.7	
Levofloxacin	abs.	*0*	0	2	18	7	0	2	0	1	0	0	0	**0**	/	/	/	/	0.063	0.125	ECOFF (0.25)	n	27	3	30
	cum %	*0.0*	0.0	6.7	66.7	90.0	90.0	96.7	96.7	100.0	100.0	100.0	100.0	**100.0**	/	/	/	/				%	90.0	10.0	
Ofloxacin	abs.	/	*0*	0	2	21	4	1	1	0	1	0	0	**0**	/	/	/	/	0.125	0.25	Not specified	n	-	-	30
	cum %	/	*0.0*	0.0	6.7	76.7	90.0	93.3	96.7	96.7	100.0	100.0	100.0	**100.0**	/	/	/	/				%	-	-	
Oxytetracycline	abs.	/	/	*0*	0	0	0	0	1	9	14	1	0	1	0	**4**	/	/	4	**128**	(T)ECOFF (16)[2]	n	25	5	30
	cum %	/	/	*0.0*	0.0	0.0	0.0	0.0	3.3	33.3	80.0	83.3	83.3	86.7	86.7	**100.0**	/	/				%	83.3	16.7	

[1]According to the specifications on the EUCAST website "Distributions and ECOFFs" (**7**).

[2]EUCAST has not defined breakpoints / cut-off values for oxytetracyline. The ECOFF of tetracycline is 8 mg/l. The (T)ECOFF of oxytetracycline can be assumed to be either 8 mg/l or 16 mg/l.

abs., absolute; cum %, cumulated frequency in percent; /, concentration not tested; S, (wildtype) susceptible; R, resistant / acquired resistance mechanism; ECOFF, epidemiological cut-off value; (T)ECOFF, tentative ECOFF

Values presented in italics indicate the number and percentage of strains that display MICs lower or equivalent to the lowest concentration tested

Values presented in bold indicate the number and percentage of strains that display MICs higher or equivalent to the highest concentration tested

Table S14: *In-vitro* activity of the tested substances against *E. cloacae* complex isolates obtained from patients with ocular surface infections (n=32)

**Substance**		**MIC (mg/l)**																	**MIC_{50} (mg/l)**	**MIC_{90} (mg/l)**	**cut-off value (mg/l)[2]**		**S**	**R**	**n**
		**≤ 0.008**	**0.016**	**0.031**	**0.063**	**0.125**	**0.25**	**0.5**	**1**	**2**	**4**	**8**	**16**	**32**	**64**	**128**	**256**	**≥512**							
Chloramphenicol	abs.	/	/	/	/	*0*	0	0	0	0	6	23	2	0	0	1	0	**0**	8	8	Not specified	n	-	-	32
	cum %	/	/	/	/	*0.0*	0.0	0.0	0.0	0.0	18.8	90.6	96.9	96.9	96.9	100.0	100.0	**100.0**				%	-	-	
Gentamicin	abs.	/	/	*0*	0	0	16	16	0	0	0	0	0	0	0	**0**	/	/	0.25	0.5	ECOFF (2)	n	32	0	32
	cum %	/	/	*0.0*	0.0	0.0	50.0	100.0	100.0	100.0	100.0	100.0	100.0	100.0	100.0	**100.0**	/	/				%	100.0	0.0	
Kanamycin	abs.	/	/	/	/	*0*	0	0	16	15	0	1	0	0	0	0	0	**0**	1	2	Not specified	n	-	-	32
	cum %	/	/	/	/	*0.0*	0.0	0.0	50.0	96.9	96.9	100.0	100.0	100.0	100.0	100.0	100.0	**100.0**				%	-	-	
Neomycin	abs.	/	/	/	/	*0*	0	25	7	0	0	0	0	0	0	0	0	**0**	0.5	1	Not specified	n	-	-	32
	cum %	/	/	/	/	*0.0*	0.0	78.1	100.0	100.0	100.0	100.0	100.0	100.0	100.0	100.0	100.0	**100.0**				%	-	-	
Levofloxacin	abs.	*0*	0	4	26	2	0	0	0	0	0	0	0	**0**	/	/	/	/	0.063	0.063	(T)ECOFF (0.125)	n	32	0	32
	cum %	*0.0*	0.0	12.5	93.8	100.0	100.0	100.0	100.0	100.0	100.0	100.0	100.0	**100.0**	/	/	/	/				%	100.0	0.0	
Ofloxacin	abs.	/	*0*	0	11	20	1	0	0	0	0	0	0	**0**	/	/	/	/	0.125	0.125	Not specified	n	-	-	32
	cum %	/	*0.0*	0.0	34.4	96.9	100.0	100.0	100.0	100.0	100.0	100.0	100.0	**100.0**	/	/	/	/				%	-	-	
Oxytetracycline	abs.	/	/	*0*	0	0	0	0	0	7	20	5	0	0	0	**0**	/	/	4	8	(T)ECOFF (16)[3]	n	32	0	32
	cum %	/	/	*0.0*	0.0	0.0	0.0	0.0	0.0	21.9	84.4	100.0	100.0	100.0	100.0	**100.0**	/	/				%	100.0	0.0	

[1]*E. cloacae* (n=26), *E. cloacae* complex (n=3), *E. hormaechei* (n=2), *E. ludwigii* (n=1)

[2]According to the specifications on the EUCAST website "Distributions and ECOFFs" (**7**).

[3]EUCAST has not defined breakpoints / cut-off values for oxytetracyline. The ECOFF of tetracycline is 8 mg/l. The (T)ECOFF of oxytetracycline can be assumed to be either 8 mg/l or 16 mg/l.

abs., absolute; cum %, cumulated frequency in percent; /, concentration not tested; S, (wildtype) susceptible; R, resistant / acquired resistance mechanism; ECOFF, epidemiological cut-off value; (T)ECOFF, tentative ECOFF

Values presented in italics indicate the number and percentage of strains that display MICs lower or equivalent to the lowest concentration tested

Values presented in bold indicate the number and percentage of strains that display MICs higher or equivalent to the highest concentration tested

Table S15: *In-vitro* activity of the tested substances against *S. marcescens* isolates sampled from patients with ocular surface infections (n=28)

Substance		MIC (mg/l)																	MIC_{50} (mg/l)	MIC_{90} (mg/l)	cut-off value (mg/l)[1]		S	R	n
		**≤ 0.008**	**0.016**	**0.031**	**0.063**	**0.125**	**0.25**	**0.5**	**1**	**2**	**4**	**8**	**16**	**32**	**64**	**128**	**256**	**≥512**							
Chloramphenicol	abs.	/	/	/	/	*0*	0	0	0	1	1	11	9	4	1	1	0	**0**	16	32	Not specified	n	-	-	28
	cum %	/	/	/	/	*0.0*	0.0	0.0	0.0	3.6	7.1	46.4	78.6	92.9	96.4	100.0	100.0	**100.0**				%	-	-	
Gentamicin	abs.	/	/	*0*	0	0	10	13	4	1	0	0	0	0	0	**0**	/	/	0.5	1	ECOFF (2)	n	28	0	28
	cum %	/	/	*0.0*	0.0	0.0	35.7	82.1	96.4	100.0	100.0	100.0	100.0	100.0	100.0	**100.0**	/	/				%	100.0	0.0	
Kanamycin	abs.	/	/	/	/	*0*	0	0	5	18	4	0	0	0	0	1	0	**0**	2	4	Not specified	n	-	-	28
	cum %	/	/	/	/	*0.0*	0.0	0.0	17.9	82.1	96.4	96.4	96.4	96.4	96.4	100.0	100.0	**100.0**				%	-	-	
Neomycin	abs.	/	/	/	/	*0*	0	16	10	1	0	0	1	0	0	0	0	**0**	0.5	1	Not specified	n	-	-	28
	cum %	/	/	/	/	*0.0*	0.0	57.1	92.9	96.4	96.4	96.4	100.0	100.0	100.0	100.0	100.0	**100.0**				%	-	-	
Levofloxacin	abs.	*0*	0	0	7	10	9	1	0	1	0	0	0	**0**	/	/	/	/	0.125	0.25	(T)ECOFF (0.5)	n	27	1	28
	cum %	*0.0*	0.0	0.0	25.0	60.7	92.9	96.4	96.4	100.0	100.0	100.0	100.0	**100.0**	/	/	/	/				%	96.4	3.6	
Ofloxacin	abs.	/	*0*	0	2	5	14	6	0	1	0	0	0	**0**	/	/	/	/	0.25	0.5	Not specified	n	-	-	28
	cum %	/	*0.0*	0.0	7.1	25.0	75.0	96.4	96.4	100.0	100.0	100.0	100.0	**100.0**	/	/	/	/				%	-	-	
Oxytetracycline	abs.	/	/	*0*	0	0	0	0	2	0	1	2	6	3	2	**12**	/	/	32	**128**	Not specified[2]	n	-	-	28
	cum %	/	/	*0.0*	0.0	0.0	0.0	0.0	7.1	7.1	10.7	17.9	39.3	50.0	57.1	**100.0**	/	/				%	-	-	

[1]According to the specifications on the EUCAST website "Distributions and ECOFFs" (**7**).

[2]Isolates of this species are regarded as resistant.

abs., absolute; cum %, cumulated frequency in percent; /, concentration not tested; S, (wildtype) susceptible; R, resistant / acquired resistance mechanism; ECOFF, epidemiological cut-off value; (T)ECOFF, tentative ECOFF

Values presented in italics indicate the number and percentage of strains that display MICs lower or equivalent to the lowest concentration tested.

Values presented in bold indicate the number and percentage of strains that display MICs higher or equivalent to the highest concentration tested

Table S16: *In-vitro* activity of the tested substances against *P. mirabilis* isolates obtained from patients with ocular surface infections (n=23)

Substance		MIC (mg/l)																	MIC_{50} (mg/l)	MIC_{90} (mg/l)	cut-off value (mg/l)[1]		S	R	n
		**≤ 0.008**	**0.016**	**0.031**	**0.063**	**0.125**	**0.25**	**0.5**	**1**	**2**	**4**	**8**	**16**	**32**	**64**	**128**	**256**	**≥512**							
Chloramphenicol	abs.	/	/	/	/	*0*	0	0	0	0	3	11	4	2	2	1	0	**0**	8	64	Not specified	n	-	-	23
	cum %	/	/	/	/	*0.0*	0.0	0.0	0.0	0.0	13.0	60.9	78.3	87.0	95.7	100.0	100.0	**100.0**				%	-	-	
Gentamicin	abs.	/	/	*0*	0	0	5	11	5	1	1	0	0	0	0	**0**	/	/	0.5	1	ECOFF (4)	n	23	0	23
	cum %	/	/	*0.0*	0.0	0.0	21.7	69.6	91.3	95.7	100.0	100.0	100.0	100.0	100.0	**100.0**	/	/				%	100.0	0.0	
Kanamycin	abs.	/	/	/	/	*0*	0	0	3	13	4	1	0	0	0	1	1	**0**	2	8	Not specified	n	-	-	23
	cum %	/	/	/	/	*0.0*	0.0	0.0	13.0	69.6	87.0	91.3	91.3	91.3	91.3	95.7	100.0	**100.0**				%	-	-	
Neomycin	abs.	/	/	/	/	*0*	0	1	10	9	1	0	1	1	0	0	0	**0**	2	4	Not specified	n	-	-	23
	cum %	/	/	/	/	*0.0*	0.0	4.3	47.8	87.0	91.3	91.3	95.7	100.0	100.0	100.0	100.0	**100.0**				%	-	-	
Levofloxacin	abs.	*0*	0	0	11	8	3	0	1	0	0	0	0	**0**	/	/	/	/	0.125	0.25	ECOFF (0.25)	n	22	1	23
	cum %	*0.0*	0.0	0.0	47.8	82.6	95.7	95.7	100.0	100.0	100.0	100.0	100.0	**100.0**	/	/	/	/				%	95.7	4.3	
Ofloxacin	abs.	/	*0*	0	0	11	9	2	0	1	0	0	0	**0**	/	/	/	/	0.25	0.5	ECOFF (0.5)	n	22	1	23
	cum %	/	*0.0*	0.0	0.0	47.8	87.0	95.7	95.7	100.0	100.0	100.0	100.0	**100.0**	/	/	/	/				%	95.7	4.3	
Oxytetracycline	abs.	/	/	*0*	0	0	0	0	0	0	0	1	0	0	0	**22**	/	/	**128**	**128**	(T)ECOFF (256)[3]	n	-	-	23
	cum %	/	/	*0.0*	0.0	0.0	0.0	0.0	0.0	0.0	0.0	4.3	4.3	4.3	4.3	**100.0**	/	/				%	-	-	

[1]According to the specifications on the EUCAST website "Distributions and ECOFFs" (**7**).

[2]Isolates of this species are regarded as resistant.

[3]EUCAST has not defined breakpoints / cut-off values for oxytetracyline. The ECOFF of tetracycline is 128 mg/l. The (T)ECOFF of oxytetracycline can be assumed to be either 128 mg/l or 256 mg/l.

abs., absolute; cum %, cumulated frequency in percent; /, concentration not tested; S, (wildtype) susceptible; R, resistant / acquired resistance mechanism; ECOFF, epidemiological cut-off value; (T)ECOFF, tentative ECOFF

Values presented in italics indicate the number and percentage of strains that display MICs lower or equivalent to the lowest concentration tested

Values presented in bold indicate the number and percentage of strains that display MICs higher or equivalent to the highest concentration tested

Table S17: *In-vitro* activity of the tested substances against *K. oxytoca* isolates obtained from patients with ocular surface infections (n=22)

**Substance**		**MIC (mg/l)**																	**MIC_{50} (mg/l)**	**MIC_{90} (mg/l)**	**cut-off value (mg/l)[1]**		**S**	**R**	**n**
		**≤ 0.008**	**0.016**	**0.031**	**0.063**	**0.125**	**0.25**	**0.5**	**1**	**2**	**4**	**8**	**16**	**32**	**64**	**128**	**256**	**≥512**							
Chloramphenicol	abs.	/	/	/	/	*0*	0	0	0	10	10	0	2	0	0	0	0	**0**	4	4	Not specified	n	-	-	22
	cum %	/	/	/	/	*0.0*	0.0	0.0	0.0	45.5	90.9	90.9	100.0	100.0	100.0	100.0	100.0	**100.0**				%	-	-	
Gentamicin	abs.	/	/	*0*	0	0	15	5	2	0	0	0	0	0	0	**0**	/	/	0.25	0.5	ECOFF (2)	n	22	0	22
	cum %	/	/	*0.0*	0.0	0.0	68.2	90.9	100.0	100.0	100.0	100.0	100.0	100.0	100.0	**100.0**	/	/				%	100.0	0.0	
Kanamycin	abs.	/	/	/	/	*0*	0	0	9	7	3	1	2	0	0	0	0	**0**	2	8	Not specified	n	-	-	22
	cum %	/	/	/	/	*0.0*	0.0	0.0	40.9	72.7	86.4	90.9	100.0	100.0	100.0	100.0	100.0	**100.0**				%	-	-	
Neomycin	abs.	/	/	/	/	*0*	1	15	6	0	0	0	0	0	0	0	0	**0**	0.5	1	Not specified	n	-	-	22
	cum %	/	/	/	/	*0.0*	4.5	72.7	100.0	100.0	100.0	100.0	100.0	100.0	100.0	100.0	100.0	**100.0**				%	-	-	
Levofloxacin	abs.	*0*	0	2	17	1	2	0	0	0	0	0	0	**0**	/	/	/	/	0.063	0.125	Not specified	n	-	-	22
	cum %	*0.0*	0.0	9.1	86.4	90.9	100.0	100.0	100.0	100.0	100.0	100.0	100.0	**100.0**	/	/	/	/				%	-	-	
Ofloxacin	abs.	/	*0*	0	3	17	1	1	0	0	0	0	0	**0**	/	/	/	/	0.125	0.125	Not specified	n	-	-	22
	cum %	/	*0.0*	0.0	13.6	90.9	95.5	100.0	100.0	100.0	100.0	100.0	100.0	**100.0**	/	/	/	/				%	-	-	
Oxytetracycline	abs.	/	/	*0*	0	0	0	0	4	13	3	1	0	0	1	**0**	/	/	2	4	(T)ECOFF (8)[2]	n	21	1	22
	cum %	/	/	*0.0*	0.0	0.0	0.0	0.0	18.2	77.3	90.9	95.5	95.5	95.5	100.0	**100.0**	/	/				%	95.5	4.5	

[1]According to the specifications on the EUCAST website "Distributions and ECOFFs" (**7**).

[2]EUCAST has not defined breakpoints / cut-off values for oxytetracyline. The ECOFF of tetracycline is 4 mg/l. The (T)ECOFF of oxytetracycline can be assumed to be either 4 mg/l or 8 mg/l.

abs., absolute; cum %, cumulated frequency in percent; /, concentration not tested; S, (wildtype) susceptible; R, resistant / acquired resistance mechanism; ECOFF, epidemiological cut-off value; (T)ECOFF, tentative ECOFF

Values presented in italics indicate the number and percentage of strains that display MICs lower or equivalent to the lowest concentration tested

Values presented in bold indicate the number and percentage of strains that display MICs higher or equivalent to the highest concentration tested

Table S18: MIC values of reference strains tested and quality control ranges defined by EUCAST

Reference strain	Antimicrobial agent	MIC (mg/L)			No. of tests out of EUCAST range	MIC (mg/L) EUCAST	
		No. of tests	Median	Range		Target	Range
S. aureus ATCC 29213 MSSA	Chloramphenicol	20	8	8-32	1	4-8	2-16
	Gentamicin		0.25	0.25-0.5	-	0.25-0.5	0.125-0.5
	Kanamycin		2	2-4	Not evaluable	Not given	
	Neomycin		0.5	0.25-1	Not evaluable	Not given	
	Levofloxacin		0.25	0.25-0.5	1	0.125-0.25	0.06-0.5
	Ofloxacin		0.5	0.5-1	-	0.25-0.5	0.125-1
	Oxytetracycline		1	0.5-1	-	0.5	0.25-1
	Cefoxitin		4	2-4	-	2	1-4
	Oxacillin		0.5	0.5-1	-	-	0.125-0.5[1]
S. aureus ATCC 43300 MRSA	Chloramphenicol	16	8	8	Not evaluable	Not given	
	Gentamicin		64	32-≥128	Not evaluable	Not given	
	Kanamycin		≥512	128--≥512	Not evaluable	Not given	
	Neomycin		64	32-64	Not evaluable	Not given	
	Levofloxacin		0.25	0.25-0.5	Not evaluable	Not given	
	Ofloxacin		0.5	0.25-0.5	Not evaluable	Not given	
	Oxytetracycline		0.5	0.5-2	Not evaluable	Not given	
	Cefoxitin		≥32	≥32	Not evaluable	Not given	
	Oxacillin		≥16	≥16	Not evaluable	Not given	
S. pneumoniae ATCC 49619	Chloramphenicol	5	2	2-4	-	4	2-8
	Gentamicin		8	8	Not evaluable	Not given	
	Kanamycin		32	32	Not evaluable	Not given	
	Neomycin		32	32	Not evaluable	Not given	
	Levofloxacin		1	0.5-1	-	1	0.5-2
	Ofloxacin		2	1-2	-	2	1-4
	Oxytetracycline		0.25	0.25-0.5	-	0.25	0.125-0.5
E. coli ATCC 25922	Chloramphenicol	15	4	4-8	-	4	2-8
	Gentamicin		0.25	0.25-0.5	-	0.5	0.25-1
	Kanamycin		2	1-4	Not evaluable	Not given	
	Neomycin		0.5	0.5-2	Not evaluable	Not given	
	Levofloxacin		0.016	0.016-0.03	-	0.016-0.03	0.008-0.06
	Ofloxacin		0.03	0.03-0.06	-	0.03-0.06	0.016-0.125
	Oxytetracycline		1	1-2	Not evaluable	Not given	
P. aeruginosa ATCC 27853	Chloramphenicol	10	256	128-256	Not evaluable	Not given	
	Gentamicin		1	1	-	1	0.5-2
	Kanamycin		≥512	≥512	Not evaluable	Not given	
	Neomycin		64	32-128	Not evaluable	Not given	
	Levofloxacin		1	1	-	1-2	0,5-4
	Ofloxacin		2	1-2	Not evaluable	Not given	
	Oxytetracycline		16	8-16	Not evaluable	Not given	
H. influenzae ATCC 49766	Chloramphenicol	7	0.25	0.06-0.5	1	0.5	0.25-1

	Gentamicin		0.25	0.125-0.5	Not evaluable	Not given	
	Kanamycin		0.5	0.5-2	Not evaluable	Not given	
	Neomycin		0.5	0.5-2	Not evaluable	Not given	
	Levofloxacin		0.016	0.016	-	0.016	0.008-0.03
	Ofloxacin		0.03	0.03	-	0.03	0.016-0.06
	Oxytetracycline		0.25	0.25-0.5	Not evaluable	Not given	

[1]The range in CLSI M100-S33 is 0.125-0.5 mg/L
